# Iron-mediated soil carbon response to water-table decline in an alpine wetland

**DOI:** 10.1038/ncomms15972

**Published:** 2017-06-26

**Authors:** Yiyun Wang, Hao Wang, Jin-Sheng He, Xiaojuan Feng

**Affiliations:** 1 State Key Laboratory of Vegetation and Environmental Change, Institute of Botany, Chinese Academy of Sciences, Beijing 100093, China; 2 University of Chinese Academy of Sciences, Beijing 100049, China; 3 Department of Ecology, College of Urban and Environmental Sciences, and Key Laboratory for Earth Surface Processes of the Ministry of Education, Peking University, Beijing 100871, China; 4 Key Laboratory of Adaptation and Evolution of Plateau Biota, Northwest Institute of Plateau Biology, Chinese Academy of Sciences, Xining 810008, China

## Abstract

The tremendous reservoir of soil organic carbon (SOC) in wetlands is being threatened by water-table decline (WTD) globally. However, the SOC response to WTD remains highly uncertain. Here we examine the under-investigated role of iron (Fe) in mediating soil enzyme activity and lignin stabilization in a mesocosm WTD experiment in an alpine wetland. In contrast to the classic ‘enzyme latch’ theory, phenol oxidative activity is mainly controlled by ferrous iron [Fe(II)] and declines with WTD, leading to an accumulation of dissolvable aromatics and a reduced activity of hydrolytic enzyme. Furthermore, using dithionite to remove Fe oxides, we observe a significant increase of Fe-protected lignin phenols in the air-exposed soils. Fe oxidation hence acts as an ‘iron gate’ against the ‘enzyme latch’ in regulating wetland SOC dynamics under oxygen exposure. This newly recognized mechanism may be key to predicting wetland soil carbon storage with intensified WTD in a changing climate.

## Abstract (web summary)

The response of soil organic carbon in wetlands to water-table decline remains uncertain. Here, the authors examine the role of iron in mediating soil enzyme activity and lignin stabilization and find that iron protecting lignin phenols in soils exposed to air acts as an iron gate against the enzyme latch.

Wetlands, covering 5–8% of the Earth’s land surface, store up to 535 Gt of carbon below ground, representing ∼30% of global terrestrial carbon pool^1^,^2^. This massive carbon stock results from outweighed primary production relative to hampered decomposition under water-logged conditions^3^. However, under land-use and climate changes^1^,^4^, about half of the global wetland area (2.7–6.4 million km^2^) has been lost due to water-table decline (WTD)^1^. Improved drainage during WTD or drought may turn wetlands from carbon sinks to sources through increasing decomposition, causing major concern for carbon sequestration and climate mitigation procedures^5^,^6^.

The long-standing view on the response of wetland soil organic carbon (SOC) to WTD focuses on the positive feedback between oxygen-enhanced extracellular enzyme activity and SOC decomposition^5^,^7^,^8^. It is shown that increased oxygen availability may substantially promote the activity of phenol oxidase^8^,^9^. As elevated phenol oxidative activity eliminates phenol compounds that are toxic to hydrolytic enzymes, oxygen exposure during wetland WTD further stimulates the degradation of cellulosic compounds via breaking the ‘enzyme latch’^8^,^10^,^11^. However, positive, neutral and negative effects of WTD on SOC decomposition have been reported^5^,^12^,^13^,^14^,^15^, suggesting that other competing or confounding mechanisms may govern SOC dynamics during wetland WTD.

Redox-induced transformation of iron (Fe) is a key biogeochemical process during wetland WTD that involves the mobilization and stabilization of carbon, nitrogen, sulfur and phosphorus^16^. Several studies have observed that the presence of ferrous iron [Fe(II)] in hypoxic peatland soils may enhance phenol oxidative activity^17^,^18^,^19^. Recently, Hall *et al*.^20^ also demonstrated that the activities of hydrolytic enzymes increased with Fe(II) under anaerobic conditions, in contrast to the ‘enzyme latch’ hypothesis. Therefore, as soluble Fe(II) is oxidized to less soluble ferric iron [Fe(III)] during wetland WTD, the decline of Fe(II) may be accompanied by reduced activities of oxidative and hydrolytic enzymes, thereby counteracting the ‘enzyme latch’ and enhancing SOC preservation. Furthermore, organic matter (OM) may sorb or co-precipitate with the newly-formed reactive Fe(III) (hydro)oxides (especially poorly crystalline Fe oxides), forming Fe–OM complexes that are chemically more stable^21^,^22^. Such Fe–OM associations are considered to play an important role in carbon preservation^21^,^23^,^24^ and are estimated to stabilize ca. 21.5% of organic carbon in sediments^25^ and ca. 37.8% of SOC in US forest soils^26^. In particular, aromatics and phenolics that accumulate in wetlands are shown to exhibit a strong affinity to Fe oxides at the redox interface^27^,^28^, potentially protected from mineralization during oxygen exposure^29^.

As the second most abundant component of terrestrial plants, lignin is made up of phenolic units whose decomposition is optimized in aerobic environments^30^,^31^. In anaerobic wetlands, lignin-derived phenolic moieties are relatively concentrated and exert an important control on enzyme activity and SOC dynamics^31^. During redox fluctuations in wetlands, lignin and Fe may undergo strong association-remobilization processes. For instance, a recent incubation study employing ^13^C-labelled lignin demonstrated that oxidation of Fe(II) in a humid tropical soil specifically suppressed lignin mineralization relative to bulk SOC likely due to the association between lignin and Fe oxides^29^. However, Fe–lignin interaction during wetland WTD remains under-investigated. A major impediment to progress is that Fe-bound lignin moieties cannot be analysed by conventional method based on copper oxide (CuO) oxidation^32^, which breaks ether linkages in the macromolecular lignin and generates identifiable phenol monomers referred to as lignin phenols^33^. As demonstrated by Hernes *et al*.^32^, sorption to Fe oxides and minerals may ‘hide’ lignin and significantly reduce the yield of lignin phenols upon CuO oxidation. Therefore, an improved method is needed to quantify Fe-bound lignin phenols in the soil matrix. Recently, Shields *et al*.^34^ observed altered yields of lignin phenols upon CuO oxidation after removing reactive Fe with dithionite from Mississippi delta sediments due to the release of Fe-bound lignin. While this method has not been further validated or applied to other systems so far, it may potentially provide new insights on Fe-lignin interaction during redox changes in wetlands.

In this study, we employ a novel approach to examine Fe–SOC interaction in response to WTD-induced redox changes in a mesocosm water-table manipulation experiment on the Qinghai-Tibetan Plateau. The experiment consists of duplicated control (with submerged soils) and WTD (−20 cm relative to control) treatments in an alpine wetland dominated by *Carex pamirensis* (cf. Wang *et al*.^35^,^36^ for details of the experimental design). After 27 months of WTD treatment, extracellular enzyme activity, SOC and lignin phenols are examined together with different Fe species in the air-exposed (0–20 cm) relative to the submerged (30–40 cm) soil layers. In particular, similar to Shields *et al*.^34^, we use dithionite to remove crystalline and amorphous Fe oxides^25^ that may associate with SOC and quantify Fe-bound lignin phenols that are subsequently exposed and amenable to CuO oxidation. This method is tested using a series of supplementary analyses and applied to the wetland soils. It is observed that up to an average of 40% of lignin phenols are ‘protected’ by reactive Fe in the subsoils and not detected by the conventional CuO oxidation method. More importantly, in line with previous findings^20^,^29^, wetland WTD not only decreases phenol oxidative activity and β-1,4-glucosidase (β-glucosidase) activity but also increases Fe-bound lignin phenols in the air-exposed soils due to Fe(II) oxidation. We hence propose that redox-induced Fe transformation may act as an ‘iron gate’ against the ‘enzyme latch’ in regulating SOC dynamics under oxygen exposure during wetland WTD. This mechanism may contribute to SOC preservation in the long term and be key to understand the contrasting responses of wetland carbon storage to WTD or drought under global changes.

## Results

### Fe species and enzyme activity

**Figure 1:**
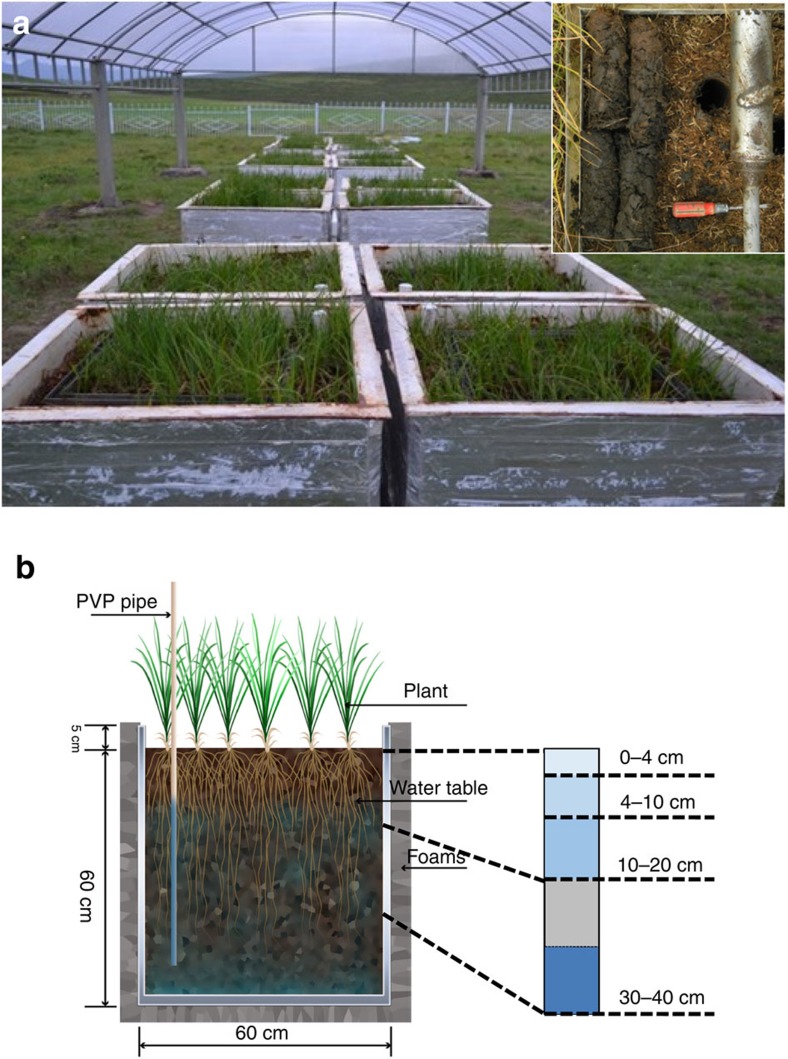
Pictures and design of the water-table decline experiment. (**a**) Picture of the mesocosm experiment with control and water-table decline treatments (*n*=4) at the Haibei Research Station, Qinghai Province, China. Picture of soil cores is shown on the upper right corner. (**b**) Cartoon diagram of the experimental design and soil sampling at different depth (modified from Wang *et al*.^36^).

**Figure 2:**
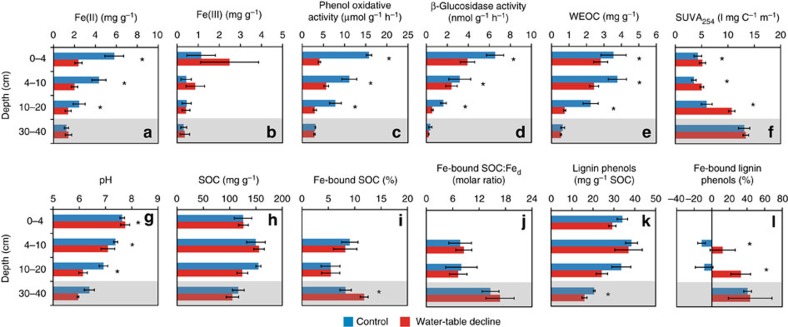
Changes in enzyme activities and soil chemical properties at different depths in the wetland water-table decline experiment. (**a**) Ferrous iron [Fe(II)]; (**b**) ferric iron [Fe(III)]; (**c**) phenol oxidative activity; (**d**) β-glucosidase activity; (**e**) water-extractable organic carbon (WEOC); (**f**) specific ultraviolet absorbance at 254 nm of WEOC (SUVA_254_); (**g**) soil pH; (**h**) soil organic carbon (SOC); (**i**) iron-bound soil organic carbon (Fe-bound SOC) calculated by equation (1); (**j**) the molar ratio of Fe-bound SOC to dithionite-extractable iron (Fe_d_); (**k**) SOC-normalized content of lignin phenols; (**l**) Fe-bound lignin phenols calculated by equation (2). The shaded soil layer (30–40 cm) was submerged under water in both treatments, whereas the other layers were air-exposed in the water-table decline treatment. Error bars represent s.e.m. (*n*=4). Asterisk denotes significant difference between control and water-table decline treatments (*P*<0.05). Note that Fe(II), Fe(III), pH and enzyme activities were measured for bulk soils whereas other properties were measured for the fine soil fraction (<53 μm). Fe-bound SOC and lignin phenols were not measured at 0–4 cm due to the limited sample size.

In the studied alpine wetland (Fig. 1), Fe(II) dominated over Fe(III), accounting for >72% of total Fe in the soil (Fig. 2a,b). Compared to the control, WTD treatment significantly decreased Fe(II) content in the air-exposed soil layers (0–20 cm; *P*<0.05), reflecting oxidative removal of Fe(II) under oxygen exposure. Interestingly, both phenol oxidative and β-glucosidase activities decreased with WTD in these layers (0–20 cm; Fig. 2c,d), accompanied by a significant decrease of water-extractable organic carbon (WEOC), an increase of its aromaticity assessed by specific ultraviolet absorbance at 254 nm (SUVA_254_) and a decrease of soil pH (*P*<0.05; Fig. 2e–g).

### Different forms of extractable Fe

Different forms of Fe oxides were examined to reveal the distribution of reactive Fe in the soil. Dithionite-extractable Fe (Fe_d_) had similar concentrations to oxalate-extractable Fe (Fe_o_) across all depths (Supplementary Fig. 1a,b), suggesting the dominance of amorphous (rather than crystalline) Fe oxides in the wetland soils. By comparison, pyrophosphate-extractable Fe (Fe_p_) had much lower concentrations (Supplementary Fig. 1c). None of these Fe contents showed any significant response to the WTD treatment due to high sample variabilities. The ratio of Fe_p_:Fe_d_, an indicator for Fe complexation degree, increased in the deeper soils (Supplementary Fig. 1d), suggesting enhanced Fe–OM complexation at depth.

### Fe-bound SOC and lignin phenols

SOC and lignin phenol contents of the fine soil fraction (<53 μm) were analysed before and after removal of crystalline and amorphous Fe oxides using the citrate–bicarbonate–dithionite (CBD) method^25^,^34^,^37^ to examine OM association with Fe oxides during WTD. Bulk SOC was overall not affected by WTD in the air-exposed or submerged soils (Fig. 2h). The dithionite treatment released significant amount of Fe-bound SOC, accounting for 5.4–11.8% of the original SOC content (Fig. 2i). The molar ratio of Fe-bound SOC to dithionite-extractable Fe (Fe_d_) ranged 7.3–16.8 (Fig. 2j) across the soil profile, suggesting that co-precipitation or complexation rather than sorption (usually with Fe-bound SOC:Fe_d_<1) dominated Fe–OM interactions in the studied wetland^38^,^39^. Notably, Fe-bound SOC was significantly higher in the submerged (30–40 cm) soil rather than in the air-exposed soils under the WTD treatment relative to the control (*P*<0.05), accompanied by a significant decrease of lignin phenols at the same depth (*P*<0.05; Fig. 2k).

Consistent with the ‘hidden lignin’ framework demonstrated by Hernes *et al*.^32^, the dithionite treatment exposed Fe-bound lignin phenols that were otherwise not detected by the CuO oxidation method, thus producing mostly positive values of Fe-bound lignin phenols (Fig. 2l). In particular, Fe-bound lignin phenols accounted for up to an average of 40% of lignin phenols in the original subsoil (30**–**40 cm; untreated with dithionite), representing a significant pool of lignin that has been underestimated in the sedimentary settings. Remarkably, Fe-bound lignin phenols were significantly higher in the air-exposed soil layers under WTD compared to the control (*P*<0.05; Fig. 2l), indicating that oxygen exposure induced stabilization of lignin in the Fe-bound form.

### OM sources in the subsoil

**Figure 3:**
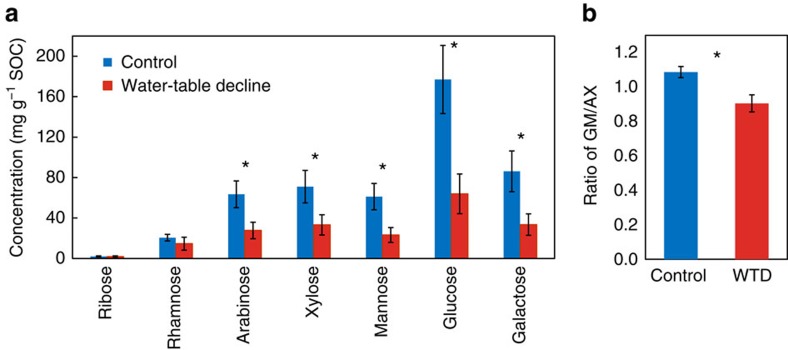
Concentrations and ratios of neutral sugars in the submerged soil. (**a**) Soil organic carbon (SOC)-normalized concentration of neutral sugars and (**b**) the ratio of (galactose+mannose)/(arabinose+xylose) (GM/AX) in the subsoils (30–40 cm; <53 μm) under the control and water-table decline (WTD) treatments. Error bars represent s.e.m. (*n*=4). Asterisk denotes significant difference between treatments (*P*<0.05).

To gain further information on the OM sources in the subsoil (30–40 cm) that witnessed a significant increase of Fe-bound SOC under WTD, we conducted a supplementary analysis of neutral sugars (that is, non-cellulosic polysaccharides), which may differentiate between microbial versus plant sources^40^,^41^. Neutral sugars amounted to 198±49 and 481±99 mg g^−1^ SOC in the WTD and control soils, respectively, representing a substantial and much larger fraction of bulk SOC relative to lignin phenols. The SOC-normalized concentration of neutral sugars decreased by ca. 50% under WTD compared to the control (*P*<0.05; Fig. 3a), echoing the response of lignin phenols. Notably, the ratio of microbial versus plant-derived sugars [(galactose+mannose)/(arabinose+xylose)]^40^ was significantly lower under WTD than the control (*P*<0.05; Fig. 3b), suggesting a preferential retention of plant-derived sugars in the WTD subsoils.

## Discussion

**Figure 4:**
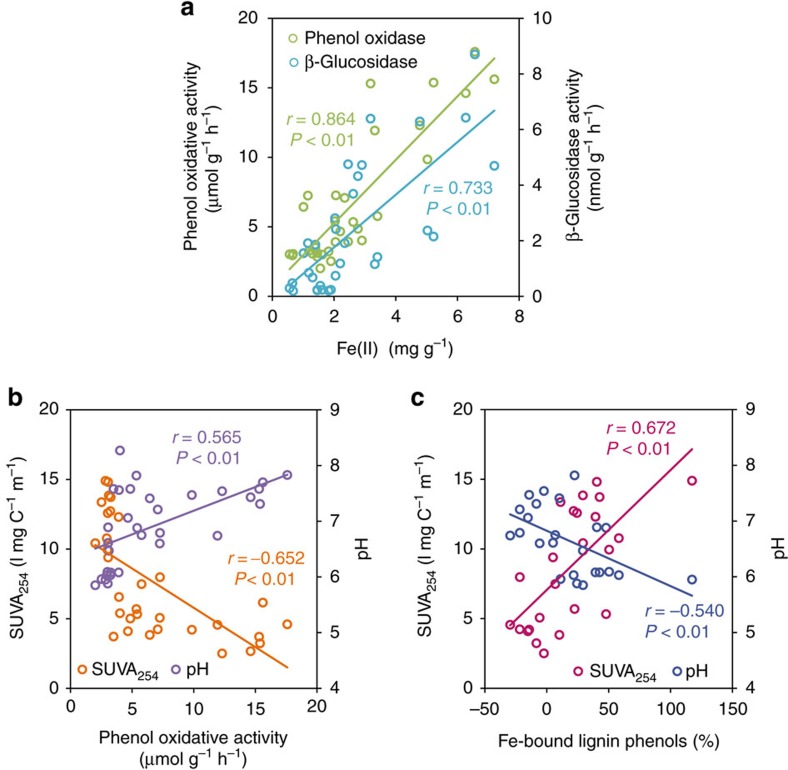
Variables influencing enzyme activities and iron-bound lignin phenols in the alpine wetland soils. (**a**) Correlations of phenol oxidative and β-glucosidase activities with ferrous iron [Fe(II)] content; (**b**) correlations of soil pH and specific ultraviolet absorbance at 254 nm (SUVA_254_) in the water-extractable organic matter with phenol oxidative activity; and (**c**) correlation between SUVA_254_ and iron-bound lignin phenol content. Solid lines indicate significant linear correlations across the entire soil profile (*n*=32 in **a**, **b** and *n*=24 in **c**).

In our mesocosm experiment, the WTD-inhibited phenol oxidative activity stands in contrast with the hypothesized opening of the ‘enzyme latch’ upon oxygen exposure^7^,^42^,^43^,^44^ but bears resemblance to a previous observation, where phenol oxidative activity increased in tandem with elevated Fe(II) and dissolved organic carbon during flooding of a paddy soil^45^. It is noteworthy that phenol oxidative and β-glucosidase activities are positively correlated with Fe(II) concentrations in our experiment (*P*<0.05; Fig. 4a), which is consistent with several previous reports^17^,^18^,^19^,^20^ and may be attributed to the following mechanisms. First, it is known that Fe(II) may enhance phenol oxidative activity by catalysing the production of hydroxyl radicals^17^,^18^,^19^. Hence, as Fe(II) concentration sharply dropped in the soils exposed to oxygen, phenol oxidative activity also declined. Second, similar to many other studies^19^,^46^,^47^, WTD decreased soil pH in the air-exposed soils (Fig. 2g) through proton release during the hydrolysis of Fe(III) produced from Fe(II) oxidation^19^. As phenol oxidative activity increases with pH in our experiment (*P*<0.05; Fig. 4b) as well as at the ecosystem level^18^, a pH decline during WTD may contribute to the inhibited phenol oxidative activity^48^. Acidification is also associated with the decreased WEOC under WTD^49^,^50^, since most soil OM has a high *p*Ka^51^, hence leading to a negative correlation between pH and WEOC in our wetland soils (*P*<0.01). As WEOC represents the most bioavailable fraction of SOC^52^, its decline may further constrain the production and activity of extracellular enzymes due to low substrate availability for soil microbes^53^,^54^. The reduced phenol oxidative activity was associated with the increased aromaticity of WEOC in the WTD-treated soils (*P*<0.05; Fig. 4b), contributing to the decline of β-glucosidase activity due to phenols’ toxicity to hydrolytic enzymes^7^,^55^.

To separate the effect of Fe(II) and pH on phenol oxidative activity, a partial correlation test was conducted. Phenol oxidative activity and Fe(II) are significantly corrrelated after the effect of pH is accounted for (*P*<0.01). In contrast, pH has no effect on phenol oxidative activity after the effect of Fe(II) content is accounted for (*P*=0.49). This result suggests that Fe(II) is the main determinant of phenol oxidative activity in our study, counteracting the positive impact of oxygen exposure during WTD. WTD may therefore have varied effects on phenol oxidative activity in wetland soils, not necessarily leading to an opening of the ‘enzyme latch’.

So where does Fe(II) play an important role in mediating the response of phenol oxidative activity to water-table variations? To answer this question, we surveyed the current literature to compare vegetation and soil characteristics of various studies that reported extracellular enzyme (mainly phenol oxidative) activities in response to WTD or drought (Supplementary Table 1). It is found that studies reporting increasing phenol oxidative activities with WTD or drought were all based on *Sphagnum*-dominated peat^5^,^8^,^10^,^56^. By comparison, WTD- or drying-induced decrease of phenol oxidative or hydrolytic enzyme activities was observed in soils dominated by non-*Sphagnum* (vascular) species (such as sedge, rice, shrub and trees)^19^,^20^,^45^,^47^,^48^,^57^. As *Sphagnum* has a much lower lignin content in their biomass as compared with vascular plants (Supplementary Fig. 2), it is reasonable to postulate that there is a lower lignin phenol content in the *Sphagnum*-dominated peat per unit of SOC relative to vascular plant-dominated soils. Varied lignin phenol contents may influence the speciation and concentration of Fe through sorption and complexation processes^27^,^58^ and thereby regulate the balance between O_2_ and Fe(II) effects on phenol oxidative activities. Alternatively, the majority (five out of seven) of the studies reporting a negative enzyme response to increasing O_2_ were based on mineral soils or organic horizons overlaying mineral soils (as is this study). The abundance or availability of Fe(II) may also play a key role in the response of extracellular enzymes to changing redox conditions. Hence, we postulate that the effect of ‘enzyme latch’ varies depending on both OM composition and soil type. The observed Fe(II) control on phenol oxidative activity is likely to be more important in mineral-rich and/or vascular plant-dominated wetland environments.

Fe did not only mediate soil carbon’s response to WTD through altering soil enzyme activities but also through changing its association with lignin. The observed increase of Fe-bound lignin phenols in the air-exposed soil layers echoes lignin stabilization with the oxidation of added Fe(II) into a moist tropical soil^29^. Since none of the extractable Fe increased under WTD (Supplementary Fig. 1), the accumulation of Fe-bound lignin phenols was likely driven by enhanced complexation (or co-precipitation) rather than sorption to Fe, consistent with the high Fe-bound SOC:Fe_d_ ratios. As aromatic and phenolic compounds have a strong affinity for hydrous oxides^23^,^28^, an increased aromaticity in the WEOC (Fig. 2e) may have promoted or reflected the stronger association between lignin and Fe under WTD. This hypothesis is confirmed by the positive correlation between Fe-bound lignin phenols and the aromaticity (SUVA_254_) of WEOC across the entire soil profile (*P*<0.05; Fig. 4c). Alternatively, Chen *et al*.^58^ showed that a pH decline may increase the amount of OM adsorbed to ferrihydrite. Hence, pH decrease in the air-exposed soils (Fig. 2g) may have also led to increased lignin complexation with Fe, reflected by the significant, negative correlation between Fe-bound lignin phenols and soil pH in our soils (*P*<0.01; Fig. 4c). Overall, our results provide clear evidence for the under-recognized role of Fe in stabilizing phenolic SOC during wetland WTD.

Last but not least, the observed increase of Fe-bound SOC at 30–40 cm was quite unexpected given that soils at this depth were continuously submerged in water in both treatments and that none of the extractable Fe oxides (Supplementary Fig. 1) or enzyme activities varied between the control and WTD treatments. However, root biomass significantly increased at 20–30 cm under WTD while remaining similar at the other depths^35^. We therefore postulate that a higher amount of root exudates migrated downward to the deeper soil in a mobile colloidal form associated with Fe^59^, thereby increasing Fe-bound SOC at 30–40 cm under WTD. Unfortunately, due to the high solubility of root exudates (such as small organic acids and sugars and so on)^60^, they are prone to loss during the CBD treatment, and hence we do not know of a method to analyse Fe-bound sugars (or organic acids) to confirm their accumulation yet. Nonetheless, the preferential retention of plant-derived sugars in the soil (Fig. 3b) somewhat agrees with our postulated increase of root-derived OM at 30–40 cm under WTD. Because Fe-bound SOC had a lower extractability of lignin phenols^32^ (and presumably sugars as well), its increase diluted lignin phenols and, to a higher extent, neutral sugars in the bulk soil at this depth. It is also possible that a higher amount of root-derived sugars were associated with Fe^61^ in the WTD subsoil, reducing their extractability and contributing to the lower concentration of neutral sugars in the hydrolysable fraction.

**Figure 5:**
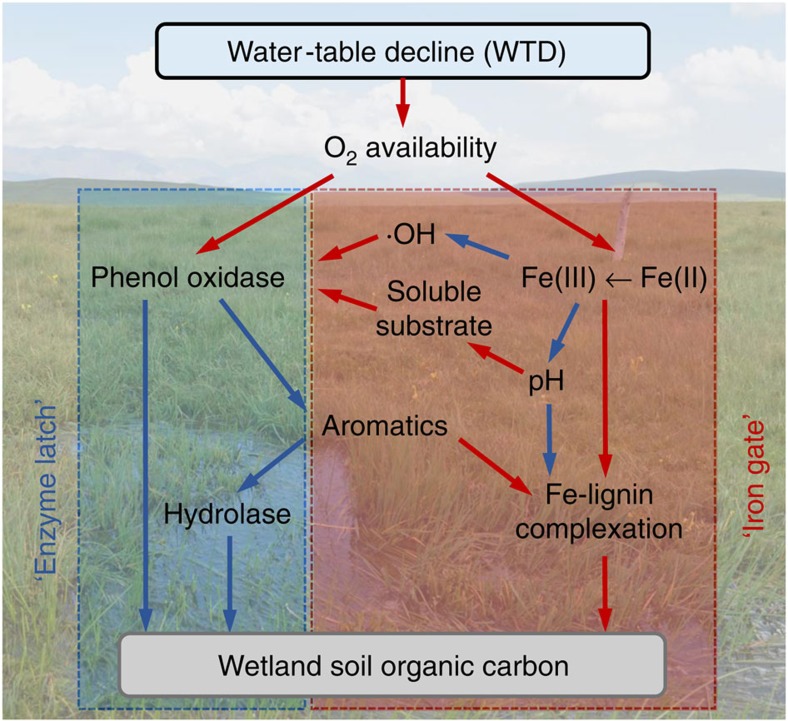
Proposed conceptual model showing the iron gate versus enzyme latch mechanisms during water-table decline. Red and blue arrows indicate positive and negative influences, respectively. O_2_-induced oxidation of ferrous iron [Fe(II)] to ferric iron [Fe(III)] decreases phenol oxidative activity through decreasing hydroxyl radicals (·OH) and reducing soluble substrates via pH decline. Reduced phenol oxidative activity leads to increased aromatic structures, which further enhances Fe-lignin complexation together with the pH decline. The ‘iron gate’ mechanism may hence lead to soil carbon stabilization under wetland water-table decline.

In summary, our innovative approach employing dithionite treatment coupled with lignin phenol analysis enabled the detection of Fe-bound lignin phenols in the wetland soil matrix, which accounted for up to an average of 40% of lignin phenols in the original subsoil. In sedimentary settings with abundant aromatic structures, Fe-bound lignin may represent a significantly underestimated pool of terrestrial carbon. Moreover, our detailed investigation on the alteration of Fe, enzyme and lignin moieties during WTD showed that oxygen exposure may not necessarily increase wetland soil carbon loss through opening the ‘enzyme latch’. In the *Carex*-dominated alpine wetland underlain by Fe-rich soils, Fe exerted a strong control on SOC dynamics by inhibiting phenol oxidative activity and promoting Fe-lignin association through Fe(II) oxidation and pH decline during WTD (the ‘iron gate’ mechanism; Fig. 5). As Fe-bound SOC moieties are considered to be more stable chemically^21^,^22^, wetland WTD may have a differential effect on the stable soil carbon pool in the longer term. Hence, redox-induced transformation of Fe plays an important and counteracting role with oxygen availability in regulating enzyme activity and SOC dynamics under wetland WTD. This newly recognized mechanism may be a key to understanding the contrasting responses of wetland carbon storage to WTD or drought in different regions, but its operating conditions and magnitude remain unclear. For instance, it is unclear if the ‘iron gate’ mechanism may outcompete ‘enzyme latch’ in the long term when reactive Fe becomes depleted or when Fe transformation slows down. Coupled Fe–SOC investigations are also needed in a wider range of wetland types to ascertain the prevalence of ‘iron gate’ in mineral-rich as well as vascular plant-dominated wetlands in order to fully assess its implications for global carbon budgets and wetland biogeochemistry in a changing climate.

## Methods

### Study site and WTD experiment

The study area is located at the Luanhaizi wetland, an alpine wetland on the northeastern edge of Qinghai-Tibet Plateau (37°35′ N, 101°20′ E, 3,250 m above sea level), with a mean annual temperature of −1.1 °C and a mean annual precipitation of 490 mm (ref. 62). The wetland is dominated by peaty soil (silty clay loam of Mat-Cryic Cambisol^62^) ranging from 0.2 to 2.0 m in depth. The average SOC and N content in the upper 0–30 cm is 21.6 and 1.2% (ref. 35), respectively. The dominating vegetation is *Carex pamirensis,* with several other species including *Carex atrofusca, Hippuris vulgaris, Triglochin palustre* and *Heleocharis* spp.

For the mesocosm experiment, 20 bottomless metal tanks with antirust paint (60 cm × 60 cm × 65 cm) were randomly embedded into the centre region of the Luanhaizi wetland in October 2010. In early November, when soils were frozen, the tanks were excavated with the bottoms sealed by painted metal plates and transported to the Haibei Alpine Grassland Ecosystem Research Station nearby. These tanks were surrounded by plastic foams to decrease energy exchange with the atmosphere. A transparent plastic canopy was built to prevent the influence of precipitation. One slim polyvinylpyrrolidone tube was setup at the corner of each box for water-table observation (Fig. 1; cf. Wang *et al*.^36^). The experiment started in mid-June of 2011, consisting of control (with submerged soils, *n*=4) and WTD treatments (*n*=4). Both treatments were automatically watered via micropumps (PULANDI 1205 Diaphragm Pump, Pulandi Machine Equipment Co., Shijiazhuang, China) connected to manostat systems using natural wetland water, which contained very low amounts of dissolved organic carbon and Fe such that their total inputs were negligible during the entire experiment (total dissolved organic carbon: ∼0.52–1.61 μg g^−1^ soil; total dissolved Fe: <0.008 μg g^−1^ soil). Water table was kept at 1.29±0.42 cm above and 23±0.83 cm below soil surfaces in the control and WTD treatment from June to September, respectively^36^. Soil surface was frozen from November to March/April and hence not monitored. The volume of soil column was not observed to change during the experiment.

### Soil sampling and preparation

In September 2013, multiple soil cores were sampled using soil corer (5 cm in diameter) from the centre of each treatment box and sliced into different depth sections. Four depths were chosen for subsequent analyses (0–4, 4–10, 10–20 and 30–40 cm) and combined accordingly. The first three layers (0–20 cm) were exposed to the air in the WTD treatment and submerged in water in the control. The fourth layer was continuously submerged in water in both treatments. Fresh soil samples were transported to the laboratory at 4 °C. An aliquot was used to determine enzyme activity and Fe species within 1 week (analytical schemes in Supplementary Fig. 3). Soil pH was measured by a pH metre with a soil:water ratio of 1:5 (w:w). The remaining soil was freeze-dried and sieved into three fractions: 0–53, 53–150 and 150–2,000 μm. The coarse fractions (>53 μm) were dominated by root debris. We hence focused on the finest fraction (0–53 μm) to explore SOC and lignin dynamics, which accounted for 40–60% of bulk soil mass (Supplementary Fig. 4). SOC was determined by Vario EL III (Elementar Analysensysteme, Hanau, Germany) elemental analyzer after fumigation with concentrated HCl^63^.

### Soil assay of extracellular enzyme activity

Potential phenol oxidative activity (phenol oxidase: EC 1.10.3.2) and β-glucosidase (EC 3.2.1.21) activity were measured according to Saiya-Cork *et al*.^64^. Specifically, ∼1 g of fresh bulk soil was homogenized with 125 ml of tris buffer (50 mM, pH 7.8) for 2.5 min with a magnetic stirrer. The resulting suspension (200 μl) was dispensed into 96-well microplates (eight replicate wells per sample per assay) while 50 μl of MUB-β-glucoside (200 μM) or L-3,4-dihydroxyphenylalanine (L-DOPA; 5 mM) solutions were added to each sample well, respectively. The microplates were incubated in the dark at 20 °C for 16 and 4 h for phenol oxidative and β-1,4-glucosidase activities, respectively. Absorbance was measured using Multi-Mode Microplate Reader (synergy Mx, BioTek Instruments Inc., USA) at 450 nm, while fluorescence was measured with excitation at 365 nm and emission at 450 nm. Enzyme activity was expressed in units of nmol h^−1^ g^−1^ dry soil or μmol h^−1^ g^−1^ dry soil.

An additional experiment utilizing surface wetland soils showed that cumulative L-DOPA oxidative products (calculated using light absorbance values) increased linearly with incubation time within 20 h (*P*<0.05; Supplementary Fig. 5). Hence, an incubation time of 16 h is reasonable for the assay of phenol oxidative activity here. Sorption of L-DOPA substrate onto mineral soils is known to bias phenol oxidative measurements^65^, rendering the absolute magnitude of phenol oxidative potentials not directly comparable among different soils. However, this mineral effect is not likely to be important in this study examining soil profiles with similar mineralogy and SOC content.

### Water-extractable OM analyses

WEOC was extracted by shaking 2.0 g of dried soil (<53 μm) in 35 ml of Milli-Q water on a shaker for 12 h. The suspension was filtered through pre-combusted 0.7-μm Whatman GF/F filters. An aliquot of the filtrate was acidified to pH <2 with HCl for WEOC measurement on multi N/C 3100 (Analytikjena, Germany) total organic carbon analyzer. Ultraviolet absorbance was also measured for acidified water extracts between 200 and 800 nm on an ultraviolet and visible spectrometer (Shimadzu UV-2550). Specific ultraviolet absorbance at 254 nm (SUVA_254_) indicating aromaticity^66^ was calculated as the decadal absorption coefficient divided by WEOC.

### Analysis of Fe and Fe-bound SOC

Soil Fe(II) and Fe(III) contents were analysed using the ferrozine-ultraviolet absorbance method^67^. Specifically, ∼0.2 g of fresh bulk soil was extracted by 5 ml of 0.5-M HCl overnight. Fe(II) was measured by absorbance at 562 nm on a UV-Vis spectrometer (Shimadzu UV-2550) after mixing with 5-mM ferrozine solution. Total Fe was first reduced using hydroxylamine hydrochloride (2%) and then measured as Fe(II) as above. Fe(III) was calculated by subtracting Fe(II) from total Fe and expressed in mg g^−1^ soil. Fe_p_ and Fe_o_ were extracted from dried soils (<53 μm), representing OM-complexed and poorly-crystalline Fe oxides, respectively^68^. Fe_p_ was extracted in 0.1 M sodium polyphosphate decahydrate at a soil:solution ratio of 1:100 (w:v) according to Mikutta *et al*.^69^. Fe_o_ was extracted with 0.2 M ammonium oxalate at pH 3, with a soil:solution ratio of 1:50 (w:v). Fe_d_ was extracted using modified CBD method^25^,^37^. The CBD supernatant was filtered through pre-combusted 0.7-μm GF/F filters, acidified to pH 2 to maintain Fe in the solution and kept in dark at 4 °C for 2 days to allow the oxidation of dithionite. Brownish precipitates were observed at this step (referred to as ‘CBD precipitates’) likely due to coagulation of OM with inorganics and was recovered using centrifugation. Fe_p_, Fe_o_ and Fe_d_ contents in the solution were determined on an inductively coupled plasma-optical emission spectrometer (ICP-OES; iCAP 6300, Thermo Scientific, USA). Soils from 0–4 cm were not included for the CBD treatment due to limited sample size.

Fe-bound SOC was determined in tandem with the CBD treatment according to Lalonde *et al*.^25^. Briefly, dried soils (<53 μm) were extracted with sodium chloride (NaCl) instead of CBD at an equivalent ionic strength as control experiments. Soil residues were rinsed with 10 ml of NaCl solution (1 M) three times, dried and HCl-fumigated before organic carbon measurement. Fe-bound SOC (in percentage) was determined as:


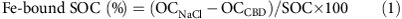


where OC_NaCl_ and OC_CBD_ are the organic carbon content of NaCl- and CBD-treated soil residues, respectively. The molar ratio of Fe-bound SOC:Fe_d_ was used as an indicator of Fe–OM interaction type^25^,^58^, which is <1.0 for sorption and >6 for co-precipitation^38^,^39^.

### Lignin analysis

Lignin phenols in the freeze-dried water-extracted soils, CBD-OM precipitates and CBD-treated residues of soils (<53 μm) were analysed using CuO oxidation method^70^,^71^. Briefly, dried soils or OM were mixed with 1 g CuO, 100 mg ammonium iron (II) sulfate hexahydrate and 15 ml of 2 M NaOH in teflon-lined bombs at 150 °C for 2.5 h. Lignin oxidation products (LOPs) were recovered from the aqueous phase with ethyl acetate after acidification with HCl (pH <2). Ethyl vanillin (3-ethoxy-4-hydroxy-benzaldehyde) and trans-cinnamic acid (3-phenyl-2-propenoic acid) were used as the recovery and internal standards, respectively. Aliquots of the LOPs were converted to trimethylsilyl derivatives and quantified on a gas chromatography-mass spectrometry (GC-MS; Thermo Fisher Scientific, USA) equipped with a DB-5 MS capillary column (30 m × 0.25 mm × 0.25 μm). Eight characteristic lignin phenols were summarized to represent SOC-normalized lignin phenol content (Λ) in the units of mg g^−1^ SOC, including vanillyl (vanillin, acetovanillone, vanillic acid), syringyl (syringaldehyde, acetosyringone, syringic acid) and cinnamyl (*p*-coumaric acid, ferulic acid) phenols.

Assuming that CBD treatment exposed Fe-bound lignin that was originally not amenable to CuO oxidation^32^,^34^, we estimated Fe-bound lignin phenols (in percentage) as:


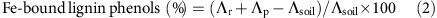


where Λ_r_, Λ_p_ and Λ_soil_ represent SOC-normalized lignin phenol concentrations in the CBD-treated soil residues, CBD-OM precipitates and water-extracted soil, respectively.

### Preliminary experiment of CBD effect on lignin phenol yield

A preliminary experiment was conducted to test whether additional lignin phenols were produced from non-Fe-bound OM following CBD treatment. Towards this end, water-soluble (that is, non-Fe-bound) plant OM was extracted from oak leaves by shaking in MilliQ water overnight, filtered through 0.45 μm and diluted to a dissolved organic carbon concentration of ∼100 mg C l^−1^. Two aliquots of the water extracts (40 ml each) were subject to CBD treatment using the same protocol as soil samples, except that dithionite was not added to one of the aliquots serving as a control. Dissolved OM in the CBD-treated solutions was recovered using C_18_ solid phase extraction cartridges and analysed by CuO oxidation method according to Louchouarn *et al*.^72^ and Spencer *et al*.^73^. As shown in Supplementary Fig. 6, both vanillyl and syringyl phenols had similar yield from the CBD-treated and control plant extracts, suggesting that CBD treatment did not cause secondary artifacts or affect the CuO oxidation efficiency for these two groups of lignin phenols. However, cinnamyl phenols decreased by ∼50% in the CBD-treated extract relative to the control, suggesting that cinnamyl phenols were subject to loss during CBD treatment and should be excluded in the calculation of Fe-bound lignin phenols. The cause for the low recovery of cinnamyl phenols remains unclear, possibly related to a lower solid phase extraction extract efficiency for the more soluble cinnamyl phenols from the aqueous phase and/or potential Fenton-like reactions with the more degradable cinnamyl phenols^74^,^75^. Regardless of the cause, our preliminary results suggest that CBD treatment is unlikely to increase the yield of CuO oxidation through producing secondary lignin phenol structures from non-Fe-bound OM. This result is echoed by the decreased yield of cinnamyl phenols but increased yields of vanillyl and syringyl phenols from the control soils after CBD treatment. Hence, we only used vanillyl and syringyl phenols for the calculation in equation (2). We also measured lignin phenols in the water extract and CBD solution of soils from different depths, which accounted for <0.4% and <5% of total lignin phenols in the original soil (Supplementary Figs 7 and 8), respectively. Soil water extract and CBD solution were hence not included in the analysis. However, it should be noted that potential loss of soluble lignin during the CBD treatment may lead to negative values of Fe-bound lignin phenols.

### Analysis of neutral sugars

Seven neutral sugars (glucose, galactose, arabinose, xylose, mannose, rhamnose and ribose) were extracted according to Spielvogel *et al*.^40^. Briefly, about 0.4 g of dried soil (<53 μm) was hydrolysed by 4 M trifluoroacetic acid at 105 °C for 4 h. Xylitol was added as a recovery standard. Neutral sugar monomers were converted to alditols and subsequently acetylated with myo-inositol added as an internal standard. Sugar monomers were quantified on a GC-MS as described previously.

### Statistics

Statistical analyses were performed using SPSS 20. Differences in SOC, enzyme activity, Fe species, lignin phenol contents and neutral sugars between control and WTD treatments were examined by two-way ANOVA with depth as a fixed effect for the air-exposed soil layers (0–20 cm) and by *t-*test for the submerged soil layer (30–40 cm). Relationships between different variables were tested by Pearson correlation tests. A partial linear correlation test was conducted to examine the effect of Fe(II) content or soil pH on phenol oxidative activity with either variable being controlled. Correlations and differences were considered to be significant at a level of *P*<0.05.

### Data availability

The data that support the findings of this study are available from the corresponding author upon request.

## Additional information

**How to cite this article:** Wang, Y. *et al*. Iron-mediated soil carbon response to water-table decline in an alpine wetland. *Nat. Commun.* **8,** 15972 doi: 10.1038/ncomms15972 (2017).

**Publisher’s note:** Springer Nature remains neutral with regard to jurisdictional claims in published maps and institutional affiliations.

## Supplementary Material

Supplementary Information
