# Supplementary material for: Iron-mediated soil carbon response to water-table decline in an alpine wetland
Source: Nat Commun. 2017 Jun 26;8:15972. doi: 10.1038/ncomms15972 (PMC5490263; doi:10.1038/ncomms15972)
Supplement: Supplementary Information [file ncomms15972-s1.pdf]

File name: Supplementary Information
Description: Supplementary Figures, Supplementary Tables and Supplementary References

Supplementary Table 1: Summary of published results on extracellular enzyme activities in response to water-table decline (WTD) or drought.

Publication	Eco-type	Dominant vegetation	Soil type	Soil pH	SOC (%)	Fe(II) (mg g^{-1} soil)	Result
Freeman et al.[1]	Peatland	*Sphagnum* and *Juncus* species	Peat	4.68−5.02	na	na	Phenol oxidative activity **increased** with O_2 (WTD or drought)
Freeman et al.[2]	Riparian gully mire	*Sphagnum*	Peat	4.5	na	na	
Fenner and Freeman[3]	Ombrotrophic peatland	*Sphagnum*	Peat	4−4.8	~50*	na	
Romanowicz et al.[4]	Oligotrophic bog	*Sphagnum*, *Polytrichum* species, sedges and ericaceous shrubs	Peat	4	na	na	
Hall and Silver[5]	Humid tropical forest	Trees (*Dacryodes excelsa, Prestoea montana, Cyrilla racemiflora*)	Ultisols, Oxisols, Inceptisols	4.3−5.3	na	0.05–7.39	Phenol oxidative or hydrolytic enzyme activity **decreased** with O_2 (WTD or drought)
Hall et al.[6]				4.3−5.3	2.8−16.7	0.11–4.11	
Liu et al.[7]	Rice paddy field	Rice (*Oryza sativa L.*)	Silt-clay Ultisol	5.9	1.1	0.2–1.7	
Toberman et al.[8]	Boreal mire peatland	Sedge, *Sphagnum* and *Juncus* species	Peat	4.0−4.7	na	na	
Toberman et al.[9]	Upland heathland	Shrub (*Calluna vulgaris L.*)	Peaty Podsol	3.9	~45*	na	
Toberman et al.[10]	Boreal mire peat	Sedge, trees, moss	Peat	4.0−5.5	na	na	
This study	Alpine wetland	Sedge (*Carex*)	Organic layers of silty clay Mat-Cryic Cambisol	6−8	7.5−18.5	0.68–7.20	

* Estimated as 50% of organic matter content. SOC: soil organic carbon; Fe(II): ferrous iron.

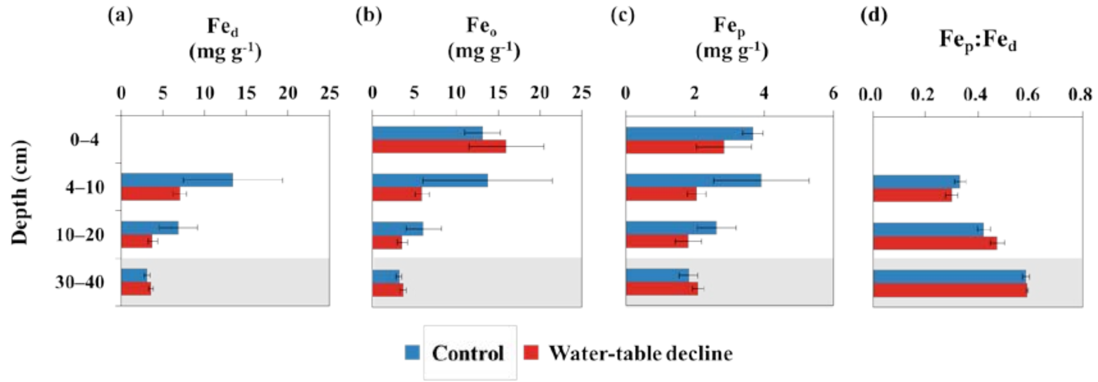

Supplementary Figure 1: Contents of different forms of extractable iron (Fe) in the wetland soils (< 53 μm). (a) Dithionite-extractable Fe (Fe_d); (b) oxalate-extractable Fe (Fe_o); (c) pyrophosphate-extractable Fe (Fe_p); (d) ratio of Fe_p:Fe_d indicating degree of Fe complexation with organic matter. Error bars represent standard error of mean (s.e.m.; n = 4). Upper- and lower-case letters denote difference among soil depths in the control and water-table decline treatment, respectively (p < 0.05). Fe_d was not measured for 0−4 cm due to the limited sample size.

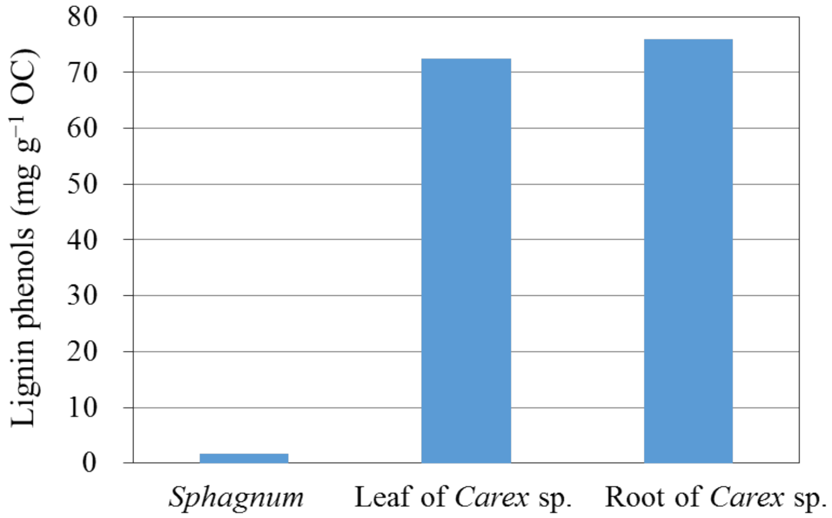

Supplementary Figure 2: Organic carbon (OC)-normalized lignin phenol concentrations in *Sphagnum* and sedge (*Carex* sp.) tissues collected from wetlands in southern China and the Haibei Station.

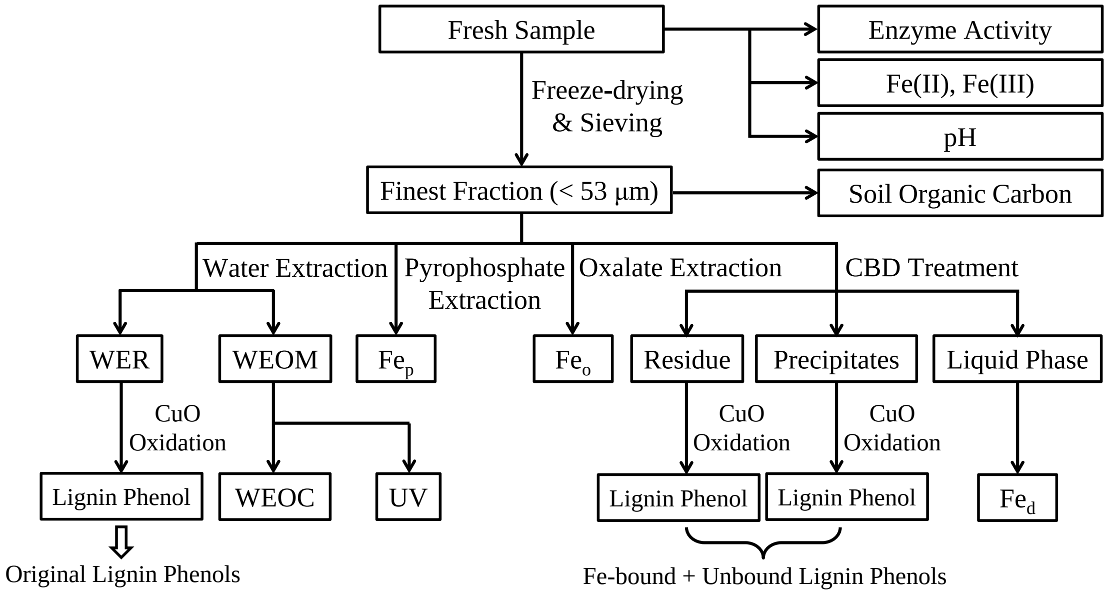

Supplementary Figure 3: Scheme of sample treatments and analyses. WER: water-extracted residue; WEOM: water-extractable organic matter; WEOC: water-extractable organic carbon; CBD: citrate-bicarbonate-dithionite method; Fe(II): ferrous iron; Fe(III): ferric iron; Fe_p: pyrophosphate-extractable iron; Fe_o: oxalate-extractable iron; Fe_d: dithionite-extractable iron.

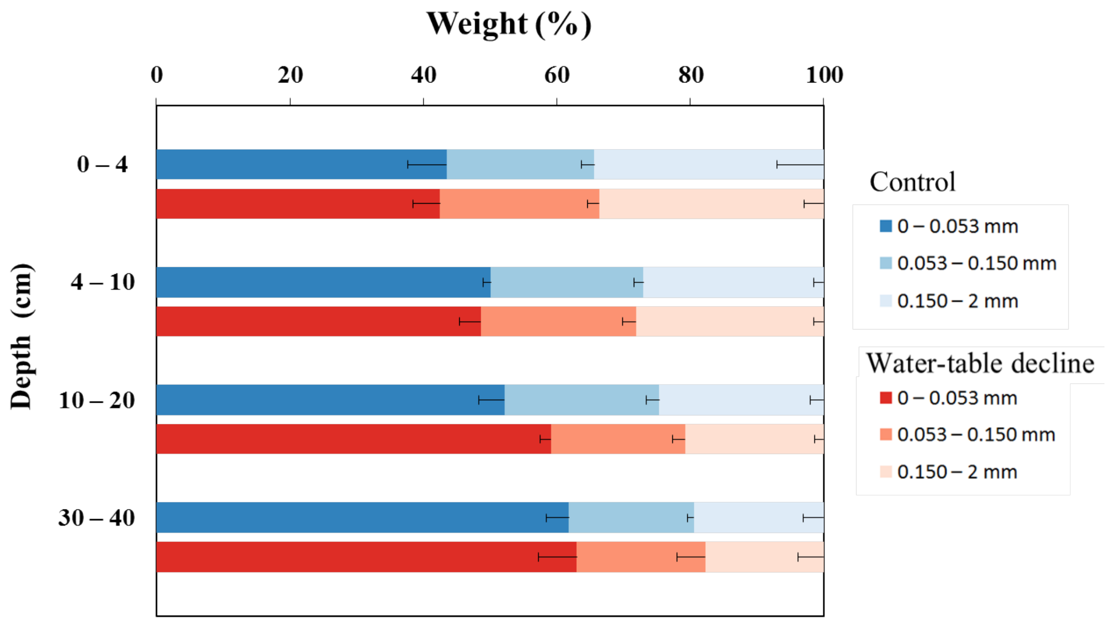

Supplementary Figure 4: Weight percentage of each grain size fraction in the control and water-table decline treated soils. Error bars represent standard error of mean (s.e.m.; n = 4).

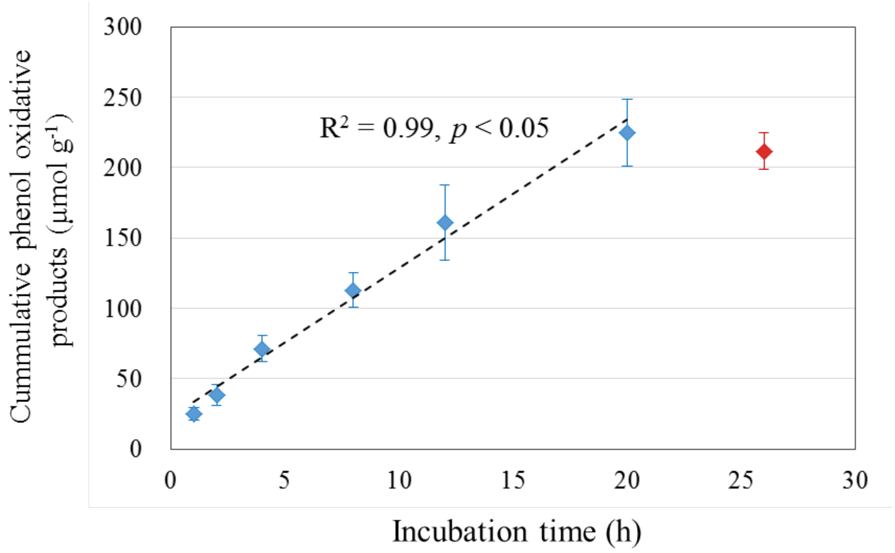

Supplementary Figure 5: Changes of cumulative phenol oxidative products in the surface wetland soil with increasing incubation time for the assay of phenol oxidative activities (mean ± s.e.m.; n = 3). Note that phenol oxidative products increased linearly with time within 20 h.

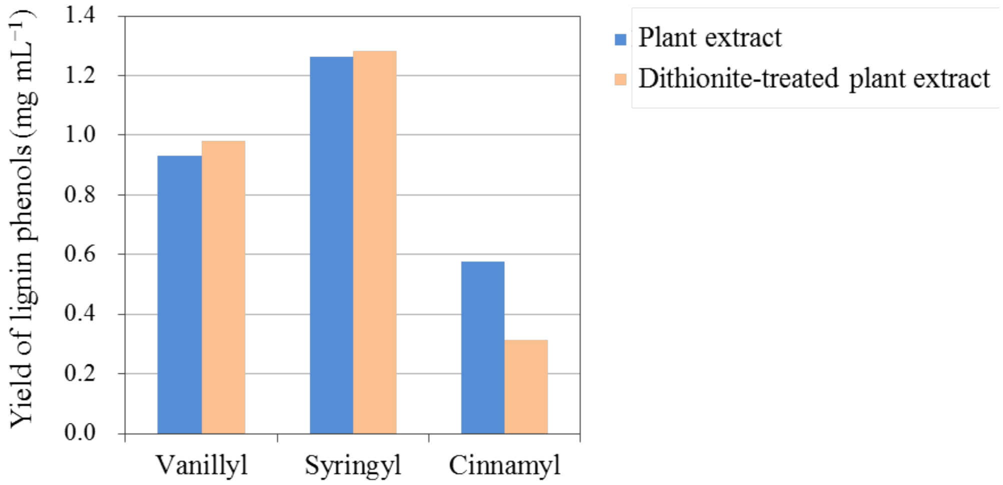

Supplementary Figure 6: Lignin phenol yields of untreated and dithionite-treated water extracts from oak leaves in the preliminary experiment.

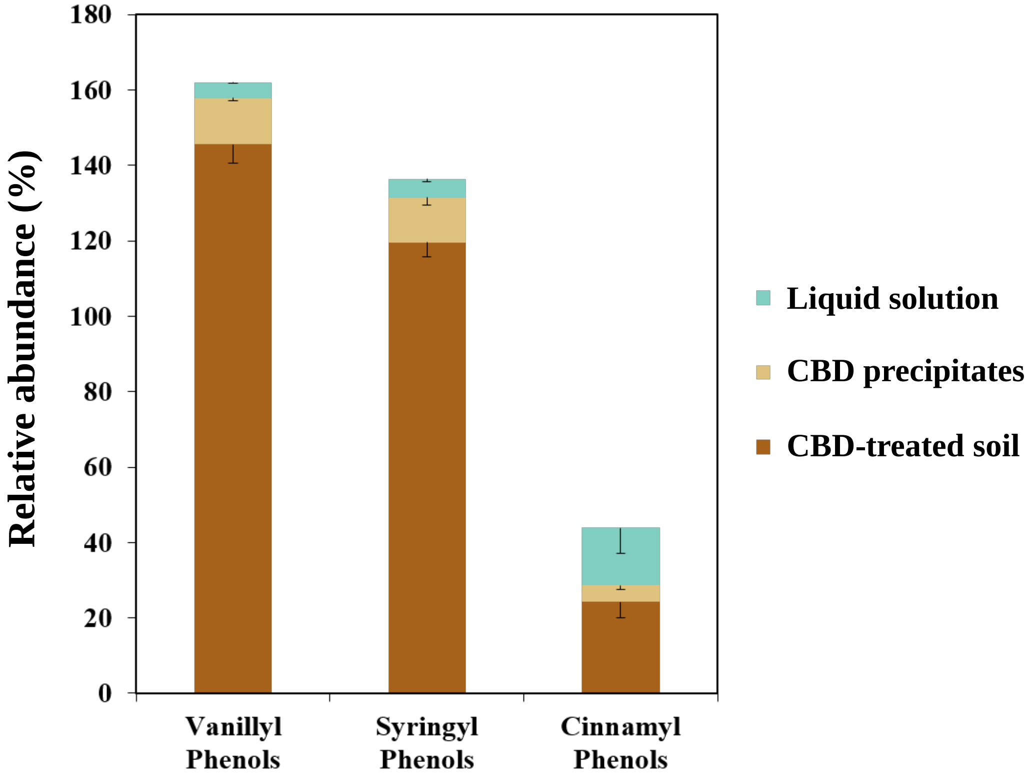

Supplementary Figure 7: Relative abundance of lignin phenols in different phases after the citrate-bicarbonate-dithionite (CBD) treatment compared with that in the original control soil at 30–40 cm. Error bars represent standard error of mean (s.e.m.; n = 4). Lignin in the liquid solution was concentrated using C_{18} solid phase extraction cartridges as described in the preliminary experiment. Note that cinnamyl phenols were relatively more abundant in the solution than vanillyl and syringyl phenols, indicating a high solubility and potential to loss during the CBD treatment.

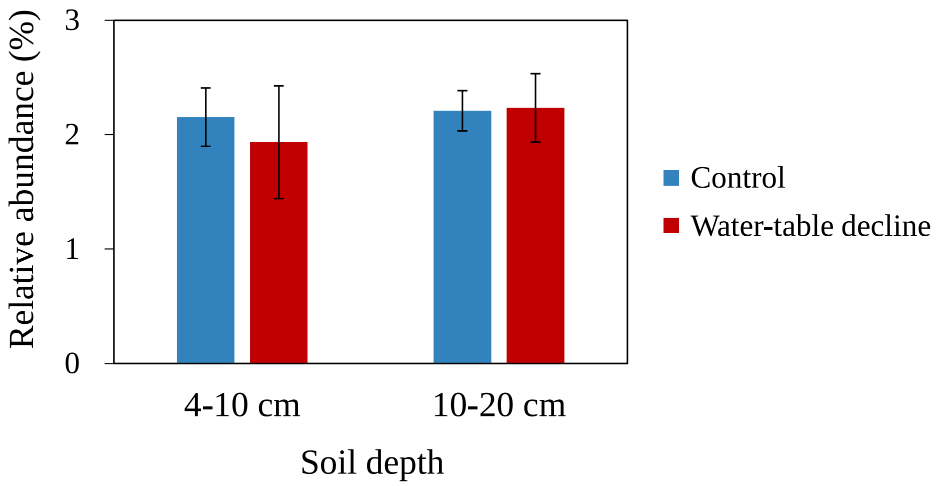

Supplementary Figure 8: Relative abundance of lignin phenols in the liquid solution after the citrate-bicarbonate-dithionite (CBD) treatment compared with that in the original soil at 4–10 and 10–20 cm in both control and water-table decline treatments. Errors represent standard error of mean (s.e.m.; n = 4).

Supplementary References

1. Freeman, C., Liska, G., Ostle, N. J., Lock, M. A., Reynolds, B. & Hudson, J. Microbial activity and enzymic decomposition processes following peatland water table drawdown. *Plant Soil* **180**, 121–127 (1996).
2. Freeman, C., Ostle, N. J., Fenner, N. & Kang, H. A regulatory role for phenol oxidase during decomposition in peatlands. *Soil Biol. Biochem.* **36**, 1663–1667 (2004).
3. Fenner, N. & Freeman, C. Drought-induced carbon loss in peatlands. *Nat. Geosci.* **4**, 895–900 (2011).
4. Romanowicz, K. J., Kane, E. S., Potvin, L. R., Daniels, A. L., Kolka, R. K. & Lilleskov, E. A. Understanding drivers of peatland extracellular enzyme activity in the PEATcosm experiment: mixed evidence for enzymic latch hypothesis. *Plant Soil* **397**, 371–386 (2015).
5. Hall, S. J. & Silver, W. L. Iron oxidation stimulates organic matter decomposition in humid tropical forest soils. *Glob. Change Biol.* **19**, 2804–2813 (2013).
6. Hall, S. J., Treffkorn, J. & Silver, W. L. Breaking the enzymatic latch: impacts of reducing conditions on hydrolytic enzyme activity in tropical forest soils. *Ecology* **95**, 2964–2973 (2014).
7. Liu, S. *et al.* Flooding effects on soil phenol oxidase activity and phenol release during rice straw decomposition. *J. Plant Nutr. Soil Sci.* **177**, 541–547 (2014).
8. Toberman, H., Freeman, C., Artz, R. R. E., Evans, C. D. & Fenner, N. Impeded drainage stimulates extracellular phenol oxidase activity in riparian peat cores. *Soil Use Manage.* **24**, 357–365 (2008).
9. Toberman, H., Freeman, C., Artz, R. R. E., Evans, C. D. & Fenner, N. Summer drought effects upon soil and litter extracellular phenol oxidase activity and soluble carbon release in an upland *Calluna* heathland. *Soil Biol. Biochem.* **40**, 1519–1532 (2008).
10. Toberman, H. *et al.* Long-term drainage for forestry inhibits extracellular phenol oxidase activity in Finnish boreal mire peat. *Eur. J. Soil Sci.* **61**, 950–957 (2010).
